# Formability Limits, Fractography and Fracture Toughness in Sheet Metal Forming

**DOI:** 10.3390/ma12091493

**Published:** 2019-05-08

**Authors:** João P. Magrinho, Maria Beatriz Silva, Luís Reis, Paulo A. F. Martins

**Affiliations:** IDMEC, Instituto Superior Técnico, Universidade de Lisboa, Av. Rovisco Pais, 1049-001 Lisboa, Portugal; joao.magrinho@tecnico.ulisboa.pt (J.P.M.); luis.g.reis@tecnico.ulisboa.pt (L.R.); pmartins@tecnico.ulisboa.pt (P.A.F.M.)

**Keywords:** sheet metal forming, formability limits, fractography, fracture toughness

## Abstract

This paper is focused on the utilisation of double edge notched tension, staggered and shear tests to determine fracture toughness and the formability limits by fracture in principal strain space. The experiments were performed in test specimens with different geometries and ligament angles, and the influence of strain hardening was taken into consideration by selecting two materials (aluminium AA1050-H111 and pure copper), with very different strain hardening exponents. Results are plotted in principal strain space, and the discussion is focused on the link between formability limits, fracture toughness and macroscopic fractography characteristics of the specimens that fail by mode I, mode II or mixed-mode.

## 1. Introduction

Failure in sheet metal forming can occur by necking, fracture or wrinkling. The formability limit by necking (also known as the forming limit curve (FLC)) was originally presented by Keeler [1] for the first quadrant of principal strain space, and subsequently extended by Goodwin [2] for the second quadrant. Failure by fracture has a relationship with the two predominant crack opening modes of fracture mechanics; By tension (mode I) and by in-plane shear or sliding (mode II) [3]. 

**Figure 1:**
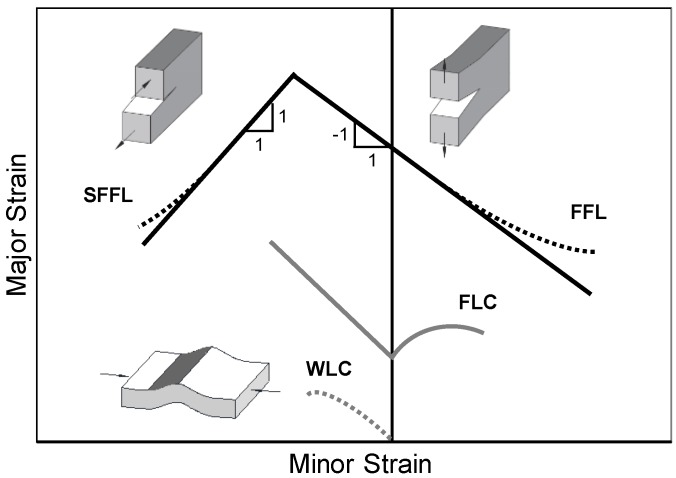
Schematic representation of the formability limits by necking, fracture (crack opening modes I and II) and wrinkling in sheet metal forming.

The fracture forming limit line (FFL) defines failure by fracture in mode I, and is plotted as a straight line falling from left to right with a slope of ‘−1’ in principal strain space. The physics of the FFL is the condition of maximum admissible thickness reduction at fracture [4]. The shear fracture forming limit line (SFFL) defines failure by fracture in mode II, and is plotted as a straight line rising from left to right with a slope of ‘+1’ in principal strain space. The physics of the SFFL is the condition of maximum in-plane shear work per unit of volume, as it was recently shown by Isik et al. [5]. Figure 1 illustrates the typical locations of the FFL and SFFL in principal strain space. 

The ‘upward curvature’ tails of the FFL and the SFFL that are plotted as dashed lines in Figure 1 are typical of materials having threshold strain values below which damage is not accumulated. This and other information on the relationship between formability limits, crack opening modes and ductile damage in sheet metal forming, is available in Martins et al. [3].

The formability limit by wrinkling in sheet metal forming is given by the wrinkling-limit curve (WLC) located in the lower left-hand of the second quadrant of principal strain space [6].

**Figure 2:**
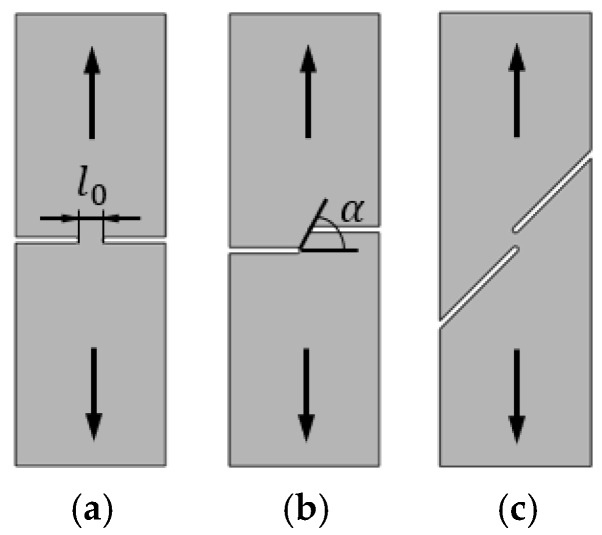
Schematic representation of (**a**) a double edge notched tensile test (DNTT) specimen [12], (**b**) a staggered DNTT specimen [13], and (**c**) a double notched shear test specimen [9].

The experimental determination of the FLC and FFL is usually performed by means of tensile, bulge and Nakajima sheet formability tests [7], whereas the determination of the SFFL is done by a variety of tests that subject material to in-plane shear loading. Isik et al. [5], for example, proposed the use of in-plane torsion tests and plane shear tests. Bao and Wierzbicki [8] suggested the utilisation of a pure shear test and a test under combined loading, both with locally reduced sheet thickness. Another test, which was originally proposed for the mechanical characterisation of thin aluminium sheets, is that based on the utilisation of double-notched shear specimens loaded in tension (Figure 2c) [9]. This test was recently used by Barnwal et al. [10] to characterise failure by the fracture of two advanced high strength steels, DP980 and TRIP1180. 

Fracture toughness is directly linked to failure by fracture in sheet metal forming, because it is the material property that accounts for the resistance of a material to crack initiation and propagation in tension or shear [11]. There are several methods to determine fracture toughness, but this paper will be focused on the essential work of fracture proposed by Cotterell and Reddel [12]. The method was originally developed for the double edge notched tension test (DNTT) that provides failure by fracture in mode I (Figure 2a). 

Later, Cotterell et al. [13] extended the essential work of fracture to staggered DNTT specimens in order to determine fracture toughness in mixed-mode consisting of opening modes I and II (Figure 2b). The fracture mixed-mode was triggered using specimens with ligament angles (stagger angles) α equal to 0, 18, 36, 54, 72 and 90°, where α=0° corresponds to mode I and α=90° to mode II.

Atkins and Mai [14] also presented experimental results for staggered DNTT specimens of steel and aluminium. They concluded that materials with moderate necking and high strain hardening, like low carbon steels, provide fracture toughness values that are independent of the ligament angle α. 

However, they also concluded that materials with low strain hardening that experience intense necking (like the aluminium B1200-H14), provide fracture toughness values that are sensitive to the ligament angle α, which is directly related to the stress state. 

The second conclusion arising from Atkins and Mai [14] is not supported by a clear tendency of the experimental data, but raises the question of the validity of using staggered DNTTs to determine fracture toughness and formability limits by fracture in principal strain space. Another key question that also arises from previous investigations is the reliability of using staggered DNTTs to produce fracture mixed-modes.

The link behind fracture toughness, macroscopic fractography and failure by fracture in principal strain space is the key motivation behind this investigation, but the two above-mentioned questions related to the uncertainty of using staggered DNTTs to determine fracture toughness and to characterise the formability limits by fracture in mixed-modes, are also important topics that will be addressed in the paper. For this purpose, the authors carried out a series of tests with DNTT specimens (Figure 2a), shear specimens (Figure 2c) and staggered DNTT specimens with different geometries, ligament sizes and angles α (Figure 2b). The influence of strain hardening was taken into consideration by using two different ductile materials, which are aluminium AA1050-H111, with very low strain hardening, and pure copper, with very high strain hardening. Formability limits by necking (FLC) give support to the presentation, but the main emphasis is put on the determination of the failure limits by fracture, namely on the utilisation of staggered DNTTs to obtain fracture strains in the mixed-mode opening conditions located in-between the FFL and the SFFL in principal strain space. 

## 2. Materials and Methods

This section presents a summary of the methods and procedures utilised in the mechanical characterisation, in the definition of the formability limits and in the determination of fracture toughness for aluminium AA1050-H111 and copper sheets with 1 mm thickness.

The aluminium alloy AA1050-H111 sheet is a common aluminium alloy for applications where moderate strength is needed, like pharmaceutical, food packaging and electronic industries. Its chemical composition is given in Table 1.

The copper sheet was cold rolled, and consists of an oxygen-free 99.9% copper (Table 1). Copper has a high ductility, and its main applications are in the production of electric cables, home appliances, and the shipbuilding industry. 

### 2.1. Material Characterisation 

The mechanical characterisation of the aluminium AA1050-H111 and copper sheets was performed by means of tensile tests on an INSTRON 5900 universal testing machine at room temperature. The specimens were cut by electrical discharge machining (EDM) out from the supplied sheets at 0, 45 and 90° degrees with respect to the rolling direction, to evaluate the influence of anisotropy. The tests followed the ASTM standard E8/E8M-16 [15], and the resulting average stress-strain curves were approximated by the power law stress-strain equation, presented in Table 2.

Table 3 provides the modulus of elasticity, the yield strength, the ultimate tensile strength, the anisotropy coefficient and the elongation at break, at 0, 45 and 90°, with respect to the rolling direction (RD) for both materials. The average values of the material properties included in Table 3 were calculated as follows (where x denotes the material property under consideration),

(1) $$\bar{x}=\frac{x_{0}+2x_{45}+x_{90}}{4}$$

### 2.2. Formability Limits

The forming limit curve (FLC) was determined by means of tensile, bulge (circular and elliptical) and Nakajima tests to cover strain loading paths from uniaxial tension to equibiaxial stretching conditions. The fracture limit by tension (fracture forming limit line (FFL)) was determined by means of the same experimental tests that were used for obtaining the FLC, plus double notched tensile tests (DNTT) to get strain values near plane strain deformation conditions. The fracture limit by in-plane shear (shear fracture forming limit line (SFFL)) was determined by means of shear tests with different ligament sizes. In addition to what was said above, staggered DNTTs with different ligament sizes $l_{0}$ and inclination angles α were performed to obtain fracture strains along the mixed-mode fracture zone located in-between the FFL and the SFFL. Table 4 summarises the experimental work plan and shows a schematic representation of the different test specimens with their geometries and dimensions. At least three repetitions were made for each formability test in order to ensure reproducibility of the results. The measurements of the specimens were performed in an optical microscope Mitutoyo model TM-505B.

The bulge and Nakajima tests were performed in a hydraulic universal testing machine ERICHSEN 145/60 and allowed the obtaining of strain loading paths from plane strain to equibiaxial stretching conditions. All the remaining tests loaded in tension were performed in the INSTRON 5900 universal testing machine that was previously utilised for the material characterisation. The Nakajima and shear tests followed the procedures and recommendations included in the ISO 12004-2 [16] and ASTM B831-11 [9] standards, respectively.

The strains at the onset of necking (FLC) were obtained by means of position-dependent [16] or time-dependent [17] methods. The position-dependent method based on circle grid analysis (CGA) was utilised in the bulge and Nakajima tests, whereas the time-dependent method, which considers the instant of time corresponding to the maximum strain rate, was utilised in all tests subjected to tensile loading.

**Figure 3:**
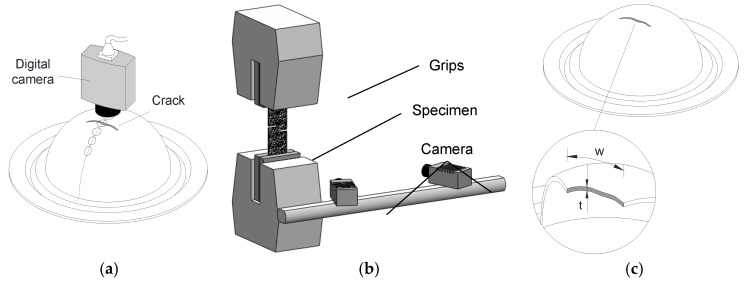
Schematic representation of (**a**) the computerised digital camera measuring system, (**b**) the digital image correlation system (DIC) and (**c**) the thickness measurements at the crack.

The Zürich n.5 procedure presented by Rossard [18], which evolved to the position-dependent method presented in the ISO standard 12004-2 [16], was utilised in CGA (refer to Figure 3a). This procedure involved electrochemical etching of a grid of circles with 2.5 mm of initial diameter on the sheet surface before deformation. The major and minor in-plane strains were measured by the computerised digital camera measuring system GPA-100 model from ASAME.

In what regards the time-dependent method proposed by Martínez-Donaire et al. [17], the determination of the maximum strain rate to identify the instant of time corresponding to the onset of failure by necking made use of the experimental strains measured with a digital image correlation (DIC) system (Figure 3b). The hardware utilised (Dantec Dynamics—model Q-400 3D) was equipped with two 6-megapixels resolution cameras with 50.2 mm focal lenses and f/8 aperture. The surface of the specimens was painted with a stochastic black speckle pattern on a uniform white background, and the correlation algorithm was the INSTRA 4D software, working with a frequency of image acquisition of 20 frames per second. A facet size of 13 pixels with a spacing grid of 7 pixels was considered.

The procedure to obtain the fracture strains required measuring the thickness of the specimens before and after deformation (Figure 3c) to obtain the ‘gauge length’ strains. The measurements of thickness after deformation were performed from individual measurements in an optical microscope Motic model BA310 MET-H. The minor strain $\varepsilon_{2}$ of the bulge and Nakajima tests was assumed to remain constant after necking, whereas that of the tests subjected to tensile loading was taken from the last measurement of the DIC system. The major strain $\varepsilon_{1}$ was obtained by incompressibility under plane strain deformation conditions $\left(d\varepsilon_{2}=0\right)$.

(2) $$\varepsilon_{1}^{f}=-\left(\varepsilon_{2}^{f}+\varepsilon_{3}^{f}\right)$$

The fracture strains of the DNTT specimens were determined by means of the procedure that had been successfully applied by Madeira et al. [19], and provided additional experimental data to construct the FFL. The fracture strains in pure shear were obtained by means of shear tests with different ligament sizes $l_{0}$ loaded in tension, and allowed, determining the SFFL and the fracture toughness in mode II. 

The staggered DNTT with different ligament sizes $l_{0}$ and angles α that was originally developed to determine fracture toughness in mixed-mode fracture consisting of opening modes I and II, was also utilised to obtain the fracture strains at the transition region between the FFL and the SFFL in principal strain space. 

The fracture surfaces of the DNTT, staggered DNTT and shear test specimens were subsequently analysed in a scanning electron microscope (SEM). The hardware utilised was the Hitachi S-2400, and the analysis allowed analysing the fractography associated to crack opening by modes I, II and mixed-mode, for each test specimen, and to link these observations with the corresponding fracture strain pairs in principal strain space.

### 2.3. Fracture Toughness

**Figure 4:**
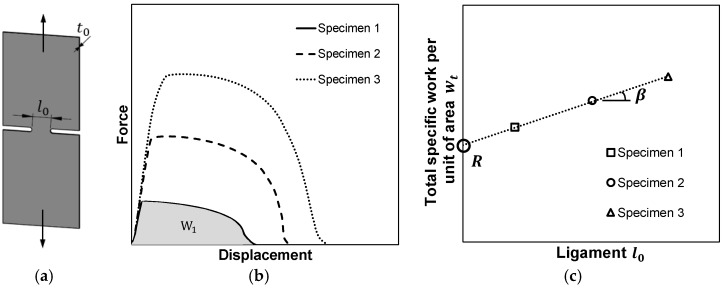
Schematic representation of the method utilised to determine fracture toughness R. (**a**) Double edge notched tension test (DNTT) specimen. (**b**) Typical force-displacement evolution for DNTT specimens with different ligaments sizes $l_{0}$. (**c**) Total specific work per unit of area $w_{T}$ and extrapolation to obtain fracture toughness R.

The determination of fracture toughness R made use of the essential work of fracture under plane stress loading conditions [12] that was originally proposed for DNTTs (Figure 4a). Application of the method requires obtaining: (i) The total work $W_{T}$, (ii) the total specific work per unit of area $w_{T}$ and (iii) the specific essential work of fracture (also known as fracture toughness, R). The total work $W_{T}$ was obtained directly from the experimental force-displacement evolution for each ligament size $l_{0}$ (Figure 4b), and corresponds to the sum of the essential work of fracture initiation and the non-essential work related to plastic deformation in the ligament area. The total specific work per unit of area $w_{T}=W_{T}/\left(t_{0}l_{0}\right)$ corresponds to the points in Figure 4c. The specific essential work of fracture (R) is calculated by extrapolating the total specific work $w_{T}$ to the limiting condition where the starting ligament length $l_{0}$ is zero, and the work related to plastic deformation is null (Figure 4c).

The method utilised to obtain fracture toughness from the DNTTs was extrapolated to the shear and staggered DNTT specimens. The shear tests allowed determining fracture toughness for crack opening in mode II (in-plane shear), and the staggered DNTTs with different ligament sizes $l_{0}$ and angles α (30, 45, 60, 70, 80 and 85°) allowed determining fracture toughness in mixed-mode fracture consisting of opening modes I and II.

## 3. Results and Discussion

This section starts by presenting the formability limits of the aluminium AA1050-H111 and copper sheets, follows with the morphology of the fractured surfaces, and ends with the determination of fracture toughness.

### 3.1. Formability Limits

The formability limits by necking (FLC) were determined by means of tensile, Nakajima and bulge tests using the methods and procedures that were previously described in Section 2.2. The fracture forming limits by tension (FFL) were determined by means of the tensile, Nakajima and bulge tests, plus the DNTTs using the methods and procedures that were also described in Section 2.2. The shear fracture forming limits (SFFL) made use of the shear tests with different ligament sizes $l_{0}$.

Table 5 presents the fracture loci equations of the FFL and SFFL for the aluminium AA1050-H111 and copper sheets. As seen, the FFL and SFFL of aluminium AA1050-H111 have slopes of −0.68 and +1.38, whereas the FFL and SFFL of copper have slopes of −0.70 and +1.41, respectively. These slopes are different from the theoretical estimates of −1 and +1, because the experimental conditions deviate from the simplifying assumptions that Isik et al. [5] used in their theoretical model. Despite these deviations, the perpendicularity between the FFL and the SFFL maintains.

**Figure 5:**
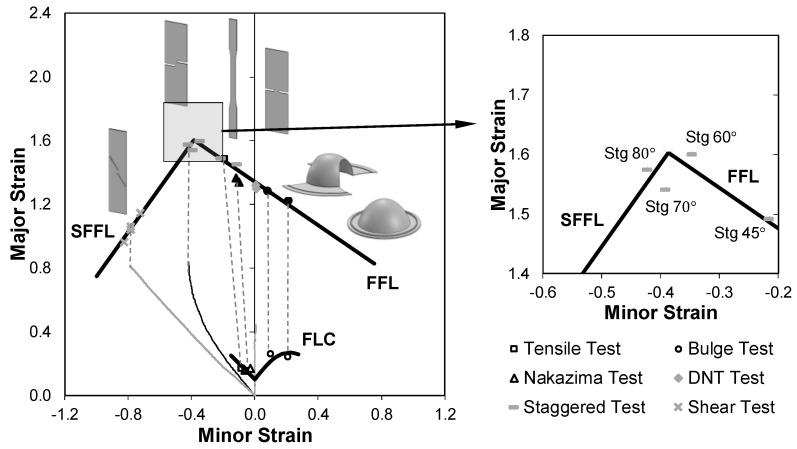
Failure loci (forming limit curve (FLC), FFL and SFFL) of aluminium AA1050-H111 sheets with 1 mm thickness. The solid and open markers correspond to strains at fracture and necking, respectively.

Figure 5 presents the formability limits and the corresponding failure strains for the aluminium AA1050-H111. The strain paths for the DNTT, staggered DNTT and shear tests were obtained by means of the DIC system, and all the fracture strain pairs were determined from measurements of the final thickness of the specimens after testing (Section 2.2). 

The staggered DNTT fracture strains pairs present smaller values of the minor strain $\varepsilon_{2}$ with the increase of the specimen’s ligament angle α until a value of 90°, which corresponds to pure shear conditions. Results of the staggered DNTT specimen with ligament angles α of 60, 70 and 80° were revealed as appropriate to characterise the transition mixed-mode fracture region located in-between the FFL and SFFL (refer to the detail in Figure 5). 

**Figure 6:**
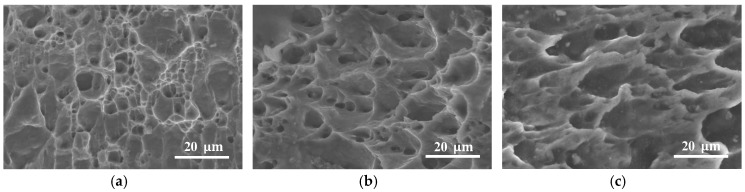
Scanning electron microscope (SEM) of the fracture surface of aluminium AA1050-H111 obtained from (**a**) DNTT (**b**) staggered DNTT with α=80° and (**c**) shear test specimens.

A fractography analysis was performed on the fracture surface of the DNTT, staggered DNTT and shear test specimens to investigate the crack opening mode and to correlate the observations with the FFL and SFFL of aluminium AA1050-H111. The SEM images of the fracture surfaces are given in Figure 6. They were obtained with a magnification of 1500×, and are representative of the entire length of the fracture surface of the specimens.

The analysis of the fracture surface of the DNTT specimen shown in Figure 6a reveals a circular dimpled structure typical of a normal fracture caused by remote loading orthogonal to the fracture surface. These results are consistent with the fracture strains of DNTT being located on the FFL (Figure 5), corresponding to the fracture forming limit by tension (mode I).

Analogously, the fracture surface of the shear test specimen shown in Figure 6c reveals elongated, parabolic dimpled structures that are different from the circular ones due to loading conditions. The open ends of the parabolic dimples are directing the shearing direction, and the overall structure is typical of fracture by sliding caused by in-plane shear. This result is consistent with the fracture strains of the shear test specimens being located on the SFFL (Figure 5) corresponding to the shear fracture forming limit (mode II).

The fracture surface of the staggered DNTT specimen shown in Figure 6b reveals a parabolic dimpled structure in-between the typical circular dimpled structure of mode I and the elongated parabolic dimpled structure of mode II. This observation allows us to consider failure by a fracture mixed-mode consisting of opening by modes I and II, which is consistent with the corresponding fracture strains being located in the transition zone between the FFL and SFFL in principal strain space. Moreover, these results are in accordance with a recent work by Gerke et al. [20], who presented an experimental SEM analysis of the fractured surfaces of a biaxial cruciform X0-specimen under proportional and non-proportional loading conditions.

**Figure 7:**
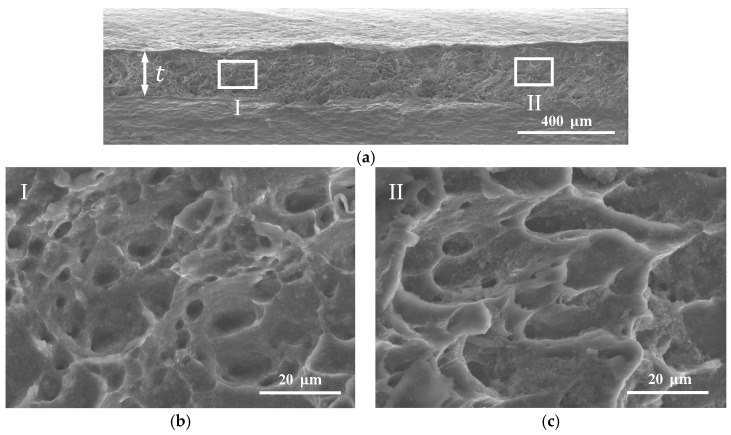
SEM of the fracture surface of an aluminium AA1050-H111 staggered DNTT specimen with α=60°. (**a**) Macro view of the fractured surface side with identification of the (**b**) tensile (mode I) and (**c**) in-plane shear (mode II) regions.

A staggered DNTT specimen with a ligament angle α=60° and a ligament size $l_{0}=8$ mm was also analysed to investigate the fracture surface of a specimen with a smaller ligament angle located in the transition zone between the FFL and SFFL (refer to the detail in Figure 5). Figure 7a presents an SEM picture of the fracture surface side with a magnification of 60× (macro view) to identify the two different locations from which the SEM magnifications of 1500× were taken (Figure 7b,c).

The first location, identified as region ‘I’ in Figure 7a, and shown with a magnification of 1500× in Figure 7b, reveals a near circular dimple-dominated structure typical of normal fracture (mode I). The second location, identified as region ‘II’ in Figure 7a and shown with a magnification of 1500× in Figure 7c, reveals an elongated dimpled structure that is characteristic of sheared fracture (mode II). This result reinforces the above-mentioned conclusion that staggered DNTT specimens of aluminium AA1050-H111 fail by fracture in mixed-mode and, therefore, are capable of providing fracture strains in the transition zone between the FFL and SFFL in principal strain space. 

**Figure 8:**
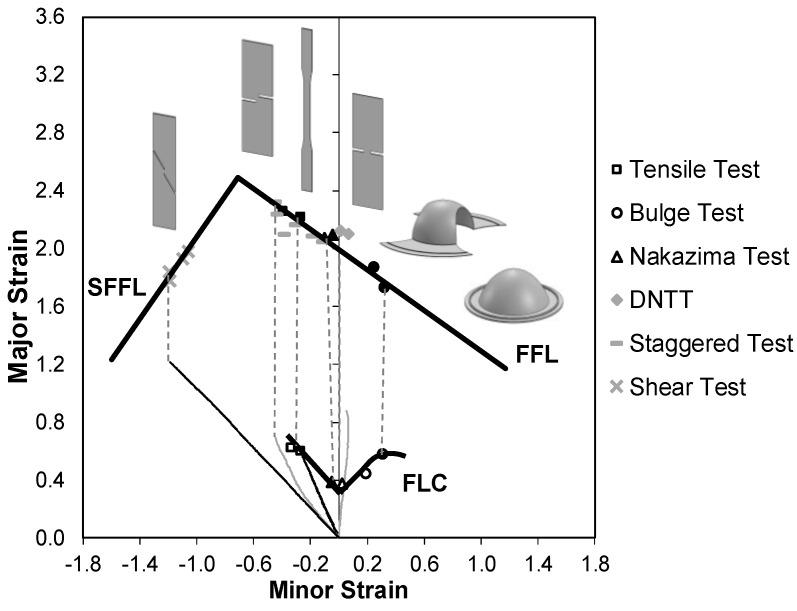
Failure loci (FLC, FFL and SFFL) of copper sheets with 1 mm thickness. The solid and open markers correspond to strains at fracture and necking, respectively.

Figure 8 presents the formability limits and the corresponding failure strains for copper in principal strain space. As expected, the fracture strains obtained from the DNTT and shear tests are located on the FFL and the SFFL, respectively. 

Just as in aluminium AA1050-H111, the fracture strains obtained for the staggered DNTTs present smaller values of the minor strain $\varepsilon_{2}$ with the increase of the ligament angle α, for the same ligament size $l_{0}$. However, and in contrast to aluminium AA1050-H111, all staggered DNTTs of copper present fracture strains on the FFL. In fact, even the specimen with α=85° provides fracture strain pairs on the FFL. 

**Figure 9:**
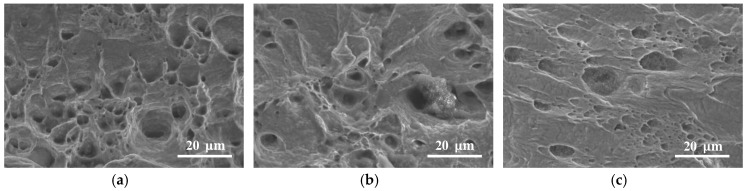
SEM of the fracture surface of copper obtained from (**a**) DNTT (**b**) staggered DNTT with α=80° and (**c**) shear test specimens.

The SEM images of the fracture surfaces of selected DNTT, staggered DNTT and shear test specimens of copper are given in Figure 9. The main conclusion arising from the observation of these images is that surface fractography fully corroborates the results plotted in principal strain space (Figure 8). In fact, the fracture surface of the DNTT and of the staggered DNTT specimens shown in Figure 9a,b reveal circular dimples typical of normal fracture (mode I), whereas the fracture surface of the shear test shown in Figure 9c reveals elongated parabolic dimples that are characteristic of sheared fracture (mode II).

**Figure 10:**
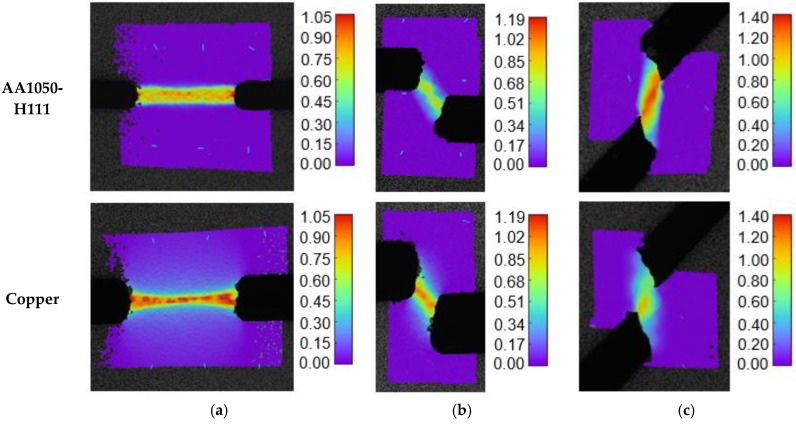
Experimental distribution of the major strain $\varepsilon_{1}$ at the onset of fracture obtained from DIC for aluminium AA1050-H111 and copper in (**a**) DNTT (**b**) staggered DNTT with α=80° and (**c**) shear test specimens.

The main difference to the results previously obtained for aluminium AA1050-H111 is that staggered DNTT specimens of copper fail by normal fracture and, therefore, are unable to provide fracture strains in the transition zone between the FFL and SFFL in principal strain space. The explanation for the different results provided by the staggered DNTTs of aluminium AA1050-H111 and copper is attributed to the fact that crack opening modes are not solely dependent on the geometry of the specimens, but also on the material properties, namely on strain hardening. This explanation is better understood by observing the experimental distributions of the major strain $\varepsilon_{1}$ at the onset of fracture for both materials, obtained from DIC (Figure 10). 

As seen in Figure 10a, the plastic deformation region of DNTT specimens of copper is wider than that of aluminium AA1050-H111, and a similar conclusion may be drawn for the staggered DNTT (Figure 10b) and shear (Figure 10c) test specimens. The consequence of the plastic deformation region being wider is two-fold. In one hand, it justifies the deviation of copper staggered DNTT specimens from pure shear conditions, because larger strain hardening coefficients n diminish localisation effects, and therefore, reduce the absolute values of strains at the onset of fracture. This justifies the reason as to why copper staggered DNTT specimens could not provide fracture strains in the transition region between the FFL and SFFL in principal strain space.

**Figure 11:**
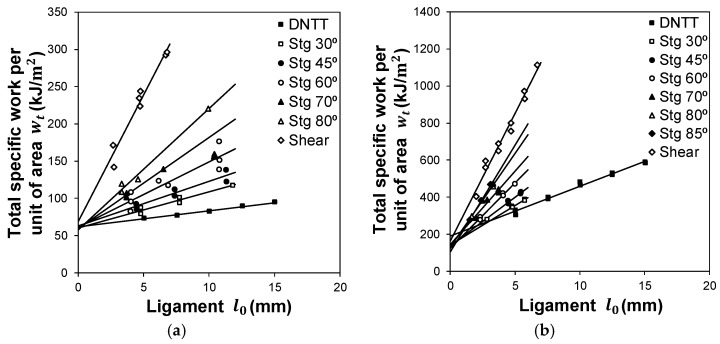
Experimental evolutions of the total specific work per unit of area $w_{T}$ with the ligament length $l_{0}$ for the DNTT, staggered DNTT with various stagger angles α and shear test specimens, in case of (**a**) aluminium AA1050-H111 and (**b**) copper.

On the other hand, it also justifies the larger slopes of the linear fittings of the total specific work per unit of area that were obtained for the copper specimens, when compared to those obtained for the aluminium AA1050-H111 specimens (Figure 11). This will be analysed in more detail during the next section of the paper focused on the determination of fracture toughness.

### 3.2. Fracture Toughness

Determination of fracture toughness R by means of the essential work of fracture (refer to the method described in Section 2.3), was successfully applied to the DNTT, staggered DNTT and shear tests that are listed at the bottom half of Table 4. Figure 11 presents the evolutions of the total specific work $w_{T}$ as function of the ligament length $l_{0}$ for aluminium AA1050-H111 (Figure 11a) and copper (Figure 11b). 

As seen, the evolutions are linear, and are in good agreement with the results obtained by Cotterell et al. [13], namely in what concerns the increase in the slope of the linear fitting of the total specific work $w_{T}$ with the specimen’s ligament angle α. This means that the slope of the total specific work increases from the DNTT specimens (α = 0°, mode I) to the shear specimens (α = 90°, mode II). 

Table 6 presents the experimental values of fracture toughness for the AA1050-H111 aluminium and copper sheets obtained by extrapolating the total specific work $w_{T}$ to the limiting condition where the starting ligament length $l_{0}$ is zero (refer to Section 2.3). 

The values obtained for aluminium AA1050-H111 can be classified into three different groups, corresponding to fracture toughness R in opening mode I ($R_{I})$, mode II ($R_{II})$ and mixed-mode ($R_{I,II})$. The highest value was found for fracture toughness in mode II $\left(R_{II}=68.07\;\mathrm{kJ}/m^{2}\right)$, but the difference to the values of $R_{I}$ corresponding to normal fracture surfaces (DNTT and staggered DNTTs with α=30 and 45°) and $R_{I,II}$ corresponding to mixed-mode fracture surfaces (staggered DNTTs with α=60 to 80°), is small. 

Moreover, the results included in Table 6 also allow a conclusion that fracture toughness $R_{I,II}$ of staggered DNTTs specimens of aluminium AA1050-H111 with α=60 to 80° is independent from ligament angle α. This result is opposite to that observed by Mai [21] for aluminium B1200-H14, which has a strain hardening coefficient n=0.045, comparable to that of aluminium AA1050-H111 (n=0.04).

In contrast, the values obtained for copper can only be classified into two groups corresponding to fracture toughness R in opening mode I ($R_{I})$ and mode II ($R_{II})$. This is because the experimental fracture strains in principal strain space (Figure 8) and the SEM images of the staggered DNTT fractured surfaces did not reveal failure by mixed-mode (Figure 9). In other words, it was not possible to determine fracture toughness $R_{I,II}$. 

This last conclusion is important, because it points out the paramount importance of combining the formability limits in principal strain space, and the SEM images to characterise the opening mode of staggered DNTT specimens. Otherwise, one may be wrongly assuming a type of fracture, when in fact it does not exist.

Under these circumstances, the main conclusion derived from the experiments with copper is that fracture toughness $R_{I}$ determined from the entire set of DNTT and staggered DNTT specimens show a tendency of diminishing with the increase in ligament angle α. The highest value $R_{I}=177.9\;\mathrm{kJ}/m^{2}$ is obtained for DNTTs (α = 0°), and the lowest value $R_{I}=105.2\;\mathrm{kJ}/m^{2}$ is obtained for the staggered DNTT with α = 85°. 

The above-mentioned results reveal some dependency on the ligament angle α, in contrast to what was found for the tests performed in aluminium AA1050-H111, and the explanation may once again be related to the differences in strain hardening (n=0.04: Aluminium AA1050-H111 and n=0.26: Copper). In fact, high strain hardening coefficients, leading to significant levels of strengthening during plastic deformation, seem to prevent mixed modes in staggered DNTTs with the staggered geometry, because smaller circular dimpled structures (typical of less ductile materials) have more difficulty evolving into elongated parabolic dimpled structures, as the stagger angle α increases more than coarser circular dimpled structures (typical of ductile materials).

## 4. Conclusions

Definition of the fracture forming limits by tension and in-plane shear in principal strain space must be accompanied by macroscopic SEM observations of the fracture surfaces, in order to validate the crack opening modes of the different tests that are used to obtain the fracture strains. The same connection is needed to associate the determination of fracture toughness to crack opening by tension, in-plane shear or mixed-mode, consisting of tension and in-plane shear.

Staggered DNTTs can be successfully utilised to characterise the transition region between the fracture forming limit line (FFL) and the shear fracture forming line (SFFL) in principal strain space, and to determine fracture toughness in fracture mixed-mode. However, precaution should be taken to avoid using results from staggered DNTTs outside their fractography domain. 

Results show that staggered DNTTs performed on aluminium AA1050-H111 are capable of covering all opening modes from tensile to in-plane shear, whereas staggered DNTTs carried out in copper with large strain hardening coefficient, are unable to deliver fracture mixed-modes. This prevents staggered DNTTs performed in materials with large strain hardening coefficients to characterise the transition region between the FFL and SFFL in principal strain space, and to deliver fracture toughness in mixed-mode. 

The differences between these two types of behaviour are attributed to the influence of strain hardening on the degree of localisation of plastic deformation around the ligament that is observed in the experimental distributions of strain obtained from DIC.

## Figures and Tables

**Table 1:** Chemical composition of aluminium AA1050-H111 and copper sheets.

Material	Chemical Composition, wt.%									
**AA1050-H111**	0.40 Fe	0.07 Zn	0.05 Cr	0.05 Cu	0.05 Mg	0.05 Mn	0.05 Ni	0.05 Ti	0.25 Si	remnant Al
**Copper**	0.0005 Bi	0.04 O	0.005 Pb	remnant Cu						

**Table 2:** Stress–strain curves for the AA1050-H111 and copper sheets.

AA1050-H111	Copper
$\bar{\sigma }=140{\bar{\varepsilon }}^{\;0.04}\;\mathrm{MPa}$	$\bar{\sigma }=427{\bar{\varepsilon }}^{\;0.26}\;\mathrm{MPa}$

**Table 3:** Mechanical properties of the aluminium AA1050-H111 and copper sheets.

Material		Modulus of Elasticity E (GPa)	$\mathrm{Yield}\;\mathrm{Strength}\;\sigma_{Y\;}(\mathrm{MPa})$	$\mathrm{Ultimate}\;\mathrm{Tensile}\;\mathrm{Strength}\;\sigma_{UTS}\;(\mathrm{MPa})$	Elongation at Break A (%)	Anisotropy Coefficient r
**AA1050-H111**	0° RD	72.7 ± 2.6	115.4 ± 0.4	119.0 ± 3.8	7.1 ± 2.1	0.71 ± 0.06
	45° RD	67.9 ± 2.9	120.4 ± 0.9	121.2 ± 0.5	5.2 ± 1.2	0.88 ± 0.10
	90° RD	71.8 ± 1.8	123.0 ± 3.6	120.8 ± 2.1	5.6 ± 1.8	0.87 ± 0.09
	**Average**	70.0 ± 7.3	119.9 ± 4.9	120.5 ± 6.4	6.8 ± 5.1	0.84 ± 0.25
**Copper**	0° RD	119.3 ± 4.3	131.2 ± 1.8	245.5 ± 1.0	34.4 ± 4.7	0.76 ± 0.08
	45° RD	115.1 ± 1.4	133.2 ± 1.5	236.2 ± 2.1	35.0 ± 3.3	1.09 ± 0.07
	90° RD	140.0 ± 4.9	141.3 ± 0.6	238.5 ± 1.1	36.3 ± 3.1	0.90 ± 0.06
	**Average**	122.4 ± 10.6	134.7 ± 3.9	239.1 ± 4.2	35.2 ± 11.1	0.96 ± 0.21

**Table 4:** Schematic representation of the experimental sheet formability tests performed in the aluminium AA1050-H111 and copper sheets.

Test		Dimensions (mm)	State of Stress	State of Strain
**Tensile**	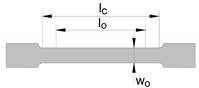	$l_{c}=80$ $l_{0}=50$ $w_{0}=12.5$	$\sigma_{1}>0$ $\sigma_{2}=\sigma_{3}=0$	$\varepsilon_{1}>0$ $\varepsilon_{2}=\varepsilon_{3}<0$
**Hydraulic bulge**	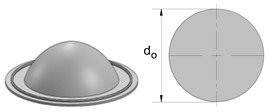	$d_{0}=175$ $d_{die}=100\;(\mathrm{Circular})$ $d_{1}:d_{2}=100:90,\;100:80\;(\mathrm{Elliptic})$	$\sigma_{1}\geq \sigma_{2}>0$ $\sigma_{3}=0$	$\varepsilon_{1}\geq \varepsilon_{2}>0$ $\varepsilon_{3}<0$
**Nakajima**	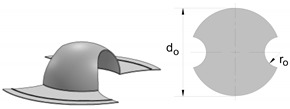	$d_{0}=210$ $r_{0}=40–80$	$\sigma_{1}>\sigma_{2}\geq 0$ $\sigma_{3}=0$	$\varepsilon_{1}\geq 0$ $-\varepsilon_{1}/2<\varepsilon_{2}<\varepsilon_{1}$ $\varepsilon_{3}<0$
**Double Notched Tension** **(DNTT)**	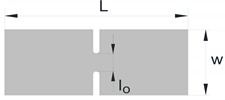	w=50 L=125 $l_{0}=5–15$	$\sigma_{1}>0$ $\sigma_{2}<0$ $\sigma_{3}=0$	$\varepsilon_{1}>0$ $\varepsilon_{2}=0$ $\varepsilon_{3}<0$
**Staggered** **DNTT**	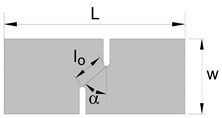	w=50 L=125 α=30–85° $l_{0}=5–12$	$\sigma_{1}>-\sigma_{2}$ $\sigma_{3}=0$	$\varepsilon_{1}>-\varepsilon_{2}$ $\varepsilon_{3}<0$
**Shear**	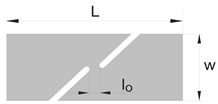	w=38.1 L=125 $l_{0}=2–6.72$	$\sigma_{1}=-\sigma_{2}$ $\sigma_{3}=0$	$\varepsilon_{1}=-\varepsilon_{2}$ $\varepsilon_{3}=0$

**Table 5:** Fracture loci for the fracture forming limit line (FFL) and shear fracture forming limit line (SFFL) of aluminium AA1050-H111 and copper sheets with 1 mm thickness.

	AA1050-H111	Copper
**FFL**	$\varepsilon_{1}+0.68\;\varepsilon_{2}=1.34$	$\varepsilon_{1}+0.70\;\varepsilon_{2}=1.99$
**SFFL**	$\varepsilon_{1}-1.38\;\varepsilon_{2}=2.14$	$\varepsilon_{1}-1.41\;\varepsilon_{2}=3.49$

**Table 6:** Fracture toughness of the aluminium AA1050-H111 and copper sheets with 1 mm thickness obtained from different tests.

Test	AA1050-H111 *R* (kJ/m^2^)	Copper *R* (kJ/m^2^)
**DNTT**	61.4 ± 0.3	177.9 ± 0.9
**Staggered 30°**	59.8 ± 0.3	142.3 ± 0.7
**Staggered 45°**	62.5 ± 0.3	137.4 ± 0.7
**Staggered 60°**	61.0 ± 0.3	124.0 ± 0.2
**Staggered 70°**	58.8 ± 0.3	144.6 ± 0.7
**Staggered 80°**	57.3 ± 0.3	131.5 ± 0.7
**Staggered 85°**	-	105.2 ± 0.5
**Shear**	68.07 ± 0.3	164.7 ± 0.8
